# Evaluating OzHarvest’s primary-school *Food Education and Sustainability Training* (FEAST) program in 10–12-year-old children in Australia: protocol for a pragmatic cluster non-randomized controlled trial

**DOI:** 10.1186/s12889-021-10302-0

**Published:** 2021-05-22

**Authors:** F. Karpouzis, R. Lindberg, A. Walsh, S. Shah, G. Abbott, J. Lai, A. Berner, K. Ball

**Affiliations:** 1 Faculty of Health, Institute for Physical Activity and Nutrition, School of Exercise and Nutrition Sciences, Deakin University, Melbourne, VIC Australia; 2 eResearch, Deakin University, Melbourne, VIC Australia; 3 School of Behavioural and Health Sciences, Australian Catholic University, Melbourne, VIC Australia; 4 Faculty of Medicine and Health, University of Sydney, Sydney, NSW Australia; 5 Intersect Australia, Sydney, NSW Australia; 6 OzHarvest, Sydney, NSW Australia

**Keywords:** Primary school, Children, Nutrition, Fruit, Vegetable, Sustainability, Food waste, Cooking, Cluster non-randomized controlled trial, Process evaluation

## Abstract

**Background:**

The promotion of healthy eating is a public health priority. Poor dietary behaviours, including low fruit and vegetable (F&V) consumption are of particular concern among children. Novel nutrition promotion strategies are needed to improve F&V consumption. Sustainability education could be used to support nutrition education within the school context. The purpose of this paper is to report the protocol for impact and process evaluation of the school-based *Food Education and Sustainability Training* (FEAST) program, designed to educate children about sustainability, food waste and nutrition, using hands-on cooking activities.

**Methods:**

A pragmatic, parallel, cluster non-randomized controlled trial with pre- and post-measures, will be implemented among 20 primary schools (10 intervention vs 10 wait-list-control) within NSW, Australia, involving children in Grades 5–6. FEAST is a curriculum-aligned program, delivered as a 1.5-h lesson/week, for a 10-week unit of inquiry, incorporating theory and cooking. FEAST was developed using theoretical frameworks which included Social Cognitive Theory and the Precede-Proceed Planning model. Primary outcomes include children’s self-reported F&V intakes (serves/day). Food literacy constructs such as: nutrition knowledge, food preparation and cooking skills, self-efficacy and behaviours, food waste knowledge and behaviours and food production knowledge, will be assessed as secondary outcomes. Process evaluation will assess program reach, adoption, implementation, maintenance, satisfaction and perceived benefits by teachers and students. An online survey (including quantitative and qualitative questions) was developed for administration at baseline (impact evaluation) and immediately post-intervention (impact and process evaluation). Intervention effects on quantitative study outcomes will be estimated with ​generalised linear mixed models, including random effects and will follow the intention-to-treat principles. Open-ended questions embedded within the surveys will be analysed qualitatively using content and thematic analyses.

**Discussion:**

Results from this trial will provide valuable information on the value of adding environmental sustainability strategies to nutrition education in schools. Results will inform the design of future research and programs focused on primary-school children’s nutrition, sustainability-related behaviours and experiential school-based interventions.

**Trial registration:**

Trial registered 14th December 2020 with the Australian and New Zealand Clinical Trials Registry (ACTRN12620001347954).

**Supplementary Information:**

The online version contains supplementary material available at 10.1186/s12889-021-10302-0.

## Background

Diet is considered to be the single most important behavioural risk factor that can be improved, to have a significant positive impact on population health [1]. Due to the continued high incidence of diet-related health problems, such as overweight, obesity, and non-communicable diseases (NCDs) among children and adults [1–3], the promotion of healthy eating continues to be a public health priority globally [3–5].

Intakes of fruits and vegetables (F&V) have become a proxy marker for healthy diets [6]. Poor dietary behaviours, that include low F&V consumption are of particular concern among children [1]. In Australia [1, 7] and internationally [8–10] children are not consuming the recommended daily servings of fruits and vegetables. Only 6.0% of Australian children consume the recommended two servings of fruit and five servings of vegetables per day [7].

Most interventions aimed at improving children’s dietary intakes have been school-based [11, 12] or community-based [12, 13]. Given that children spend most of their formative years in school, and many of their nutrition behaviours are influenced by, and established within this environment [14, 15], schools have become an obvious setting for implementation of interventions to improve the consumption of F&V [14]. As a result, teachers have become the key agents for promoting health and nutrition within schools [16].

Systematic reviews examining nutrition education programs have revealed that school-based interventions produced mixed results, with small to moderate increases in F&V intakes among children [11, 14, 17–21], with experiential learning strategies (such as school gardens, cooking skills, or food preparation) being associated with the largest effects [16]. Although school-based interventions offer a unique opportunity to educate children in health promoting activities, children’s dietary intakes still remain suboptimal [22] and more research is needed to identify novel, scalable nutrition promotion strategies, to improve F&V consumption [23–25].

The EAT-Lancet report argued that health and environmental sustainability considerations could be used to support nutrition education within the school context [26]. Growing evidence suggests that dietary patterns with low environmental impacts are also compatible with good health outcomes [27]. As such, sustainable food initiatives are accompanying nutrition education and health promotion programs in some school settings, such as the: *Farm-to-School* [28] and *Healthy Planet, Healthy Youth* [29] programs in the US; the *Farm to Cafeteria* program in Canada [30, 31]; and the *Food for Life* program in the UK [32, 33].

An evaluation of the *Healthy Planet, Healthy Youth* program found that it did not produce significant increases in F&V consumption among students compared to control schools [29]. However, the *Food for Life* program, revealed that students were significantly more likely to consume more servings of F&V, than students in comparison schools [32, 33]. The *Farm to Cafeteria* program which was evaluated by project teams involved in the funding and implementation of the program found that it increased preferences towards, consumption of a greater variety of, and willingness to try new F&V, among students [31]. A 2008 review (*n* = 15), found that students participating in the *Farm-to-School* program increased their F&V intakes, however, authors noted only one study came from a peer-reviewed journal, with very few assessments involving control groups or statistical analyses [34]. In a current systematic review of the *Farm-to-School* program (*n* = 21), authors concluded that increases in F&V consumption among students was unclear [35], which is at odds with the earlier review. One major limitation, identified by those review authors, was the failure to quantify that program’s fidelity, leading to questions about the feasibility of incorporating the *Farm-to-School* program within the classroom curricula [35].

Despite the promise of sustainable food education initiatives, there remains relatively little empirical evidence of the effectiveness of such programs, nor their acceptability among children and teachers. This would suggest that further evaluative research is needed in programs that integrate sustainable food initiatives alongside nutrition education within the school context. To our knowledge, ours is the first study to evaluate an established nutrition and sustainability education program that includes experiential activities. The *Food Education and Sustainability Training* (FEAST) program incorporates all three of these components: nutrition; sustainability; and experiential activities. FEAST is also focused on food waste, a key component of sustainability education [36] and includes food waste awareness (local and global), ways to reduce food waste, and ways to rescue foods (that would otherwise be wasted and how to turn them into new repurposed recipe ideas). (Refer to Additional file 1 for comparisons between the components of FEAST and the sustainable food initiatives discussed above.)

This trial will evaluate OzHarvest’s FEAST program in primary schools in one Australian state i.e. New South Wales (NSW). FEAST was launched in 2018 and since then has been delivered in over 170 primary schools (*n* = 8749 students) across all six Australian states, as well as the Australian Capital Territory. FEAST was designed to educate children about sustainability, food waste and nutrition, using hands-on cooking and inquiry-based learning.

This paper presents the protocol for the impact and process evaluation of the FEAST program. The primary objective of this trial is to assess the immediate effectiveness of the program on increasing F&V consumption among primary-school-aged children. The secondary objectives of this trial, will be to assess F&V variety intakes and the following food literacy constructs: nutrition knowledge, food preparation and cooking skills, self-efficacy and behaviours (i.e. preparing food, following recipes, and frequency of cooking dinner); food waste knowledge and behaviours (i.e. willingness to eat ‘imperfect’ F&V, and daily food lunch box waste behaviours); as well as food production knowledge (i.e. understanding the ‘farm to plate’ concept). Furthermore, a process evaluation will be conducted to assess program: reach (students and teachers); adoption (by schools); implementation (training of teachers, adherence by students and teachers, facilitators and barriers); maintenance (intention by students and teachers); satisfaction (of students and teachers); and perceived benefits (by teachers for their students).

## Methods

The study methods for Version 1 of this protocol (as of 22nd December 2020), will be reported in accordance with the SPIRIT (Standard Protocol Items for Clinical Trials) guidelines [37]. Ethics approval has been obtained from an Australian University Human Ethics Advisory Group (HEAG-H 31_2020) as well as from the NSW Department of Education (SERAP 2019163). The FEAST trial has been registered with the Australian and New Zealand Clinical Trials Registry (ACTRN12620001347954). Principals and teachers will provide written informed consent and parents will provide opt-out consent, if they do not wish for their child to participate in the collection of data during the implementation of the FEAST program by OzHarvest.

### Trial design

This study will employ a pragmatic approach, involving a parallel, cluster, non-randomized controlled trial (NRCT) with pre- and post-measures. It will involve 20 primary schools (10 intervention vs 10 wait-list control) with children in Grades 5–6, aged 10–12 years, within NSW, Australia. Schools that have self-selected to undertake the FEAST program in 2021 will be invited to participate in this trial.

OzHarvest, a not-for-profit community-based, food-rescue organization, has designed the FEAST program and has been training teachers to implement the program within the classroom setting, in Australian primary-schools, since 2018. The intervention runs for one school term, approximately 10 weeks. Schools participating in the FEAST program during Term 2 (19 April-25 June) of the 2021 scholastic school year will be invited to act as the intervention schools for this trial. Pre- and post-intervention surveys will be issued in the first and last weeks of term, respectively.

Schools participating in the FEAST program during Term 3 (12 July-17 September) and Term 4 (5 October-17 December), will be invited to act as the comparator group (i.e. wait-list control schools). The wait-list control schools will continue with their usual academic programs and will complete the surveys at the same time as the intervention schools (i.e. during the first and last weeks of Term 2). When the wait-list control schools undertake the FEAST program during their chosen school term (Terms 3 or 4), they will be issued with the post-intervention surveys again (third data collection point [T3]), only if they are willing to complete the surveys for their own interest. Previous experience suggests obtaining a third wave of follow-up data from schools is challenging and hence this will not be compulsory. Given variations in the timing of administration and likely low response rates, these data will be used for school feedback only, and not included in the trial.

### Study setting and eligibility criteria

Government and non-government primary schools in urban or rural locations in the state of NSW, in Australia, who have registered to undertake the FEAST program in 2021, will be eligible to participate in this trial. Students in Grades 5, 6 or composite Grade 5/6 classes (combined Grade 5 and 6), who have access to a school computer and a school email address, will be eligible to participate. Schools that cater exclusively to children with special needs or particular health conditions and/or schools that have already undertaken the FEAST program previously, will be excluded from trial participation.

### Intervention

The FEAST program was developed through a collaborative process involving OzHarvest staff (with backgrounds in education, nutrition, and sustainability) with inputs from the education sector (i.e. primary school teachers interviewed in focus groups to provide feedback on the program). It incorporated both the Precede-Proceed Planning model (PPM) [38] and Social Cognitive Theory (SCT) [39]. The PPM incorporates an ecological approach to an intervention which includes planning and evaluation [38]. This model is widely used in health promotion, as it acknowledges individual health behaviour, as well as community needs and wants [38, 40]. Guided by SCT [41], the FEAST program components were designed based on concepts of behavioural capability, outcome expectations, self-efficacy, and observational learning [39]. The use of observational learning or role modelling concepts will be incorporated into classroom cooking activities which are designed to be facilitated by teachers, parents/caregivers, and/or community volunteers that will participate in the practical components of the program. Curricular components include activities that target knowledge and skills with the intent to change behaviour [39].

FEAST is a primary-school, classroom-based, curriculum-aligned program, that uses inquiry-based approaches to learning, which are student-centred and interactive and recommended by the NSW Department of Education as a strategy that can positively impact student learning outcomes [42]. Inquiry-based learning focuses on investigation and problem-solving, and when applied to primary education to stimulate scientific inquiry, has been found to produce positive feedback from students, as it increased interest and motivation in science classes [43].

OzHarvest provides training, resources and support to teachers to deliver a recommended 1.5-h lesson/week, for a 10-week unit of inquiry (incorporating 10 theory and six practical activities) within the classroom setting. The FEAST program has been integrated with lessons mapped to the Australian Curriculum, embracing Grade 5–6 key learning areas (KLAs) i.e. English [44]; STEM (Science, Technology, Engineering, and Mathematics) curriculum [45]; general capabilities (Literacy, Numeracy, Information and Communication Technologies, Critical and Creative Thinking, Personal and Social Capability and Intercultural Understanding) [46]; and the cross-curriculum priority of sustainability [47]. FEAST has been aligned with the Australian Dietary Guidelines and state/territory-based healthy eating strategies [1], with all recipes including either fruits and/or vegetables (which are also the two most wasted food groups [48]). The program includes a range of educational resources designed to assist in the delivery of the theory and practical components as outlined in Additional file 2 [49]. The lesson plan topics are outlined in Additional file 3 and the practical guide components are outlined in Additional file 4.

Grade 5–6 classroom teachers from the schools that volunteer to participate in the FEAST program, will be invited for a training workshop at OzHarvest offices. A Professional Learning Program will be delivered over six hours of face-to-face training. During the training day, two FEAST education team members will train the teachers in food preparation and cooking skills (making the hot and cold recipes designed for the FEAST program). To assist the teachers and students during the class practical components, teachers will be provided with instructions and resources to seek volunteers throughout the school community such as parents/caregivers, grandparents, and/or school staff etc. Trained volunteers will also be available through OzHarvest, to assist during the practical components. It is recommended that the teachers organize at least one adult per five students, for each of the practical sessions, in addition to themselves.

A 3-h NESA accredited online teacher training version of the FEAST program was developed for schools in regional and remote areas of NSW (as defined in the *‘school facts location identified’*), which is published on the *MySchool* website [50]. This can also be used by teachers that are unable to attend the 6-h face-to-face training, due to additional circumstances, including COVID19 restrictions.

The teachers using the online training have the option to work through the training module at their own pace. Teacher instructions on how to use the online learning resources and adapt them to their students’ needs have also been included in the online training module. Lesson plans; how to conduct the practical cooking sessions with instructional videos on good cooking techniques for making some of the recipes at home, including parent roles; and access to all of the same resources as the face-to-face training are available through the online module. Email and phone support from the FEAST education team will also be available to the teachers undertaking the online training. The online training will also be used in the event that COVID19 restrictions prevent face-to-face training in 2021.

Strategies to improve adherence to the FEAST program include: accredited teacher training program; a complete curriculum package for the teachers; lessons plans mapped to the Australian Curriculum, KLAs (English and STEM), general capabilities, and cross-curriculum priority area of sustainability; activity booklets for students with answer booklets for teachers; training of OzHarvest volunteers to assist in the practical activities (subject to COVID19 regulations); as well as open communication (phone and/or email) between the FEAST education team and teachers during implementation, to provide support.

Strategies to support data collection involve: training the teachers to use the REDcap platform (used for data collection) and to familiarise them with survey questions; training OzHarvest volunteers to assist in data collection; a video by Ronni Kahn (OzHarvest CEO and founder) thanking students and teachers for participating in the FEAST program and encouraging them to complete the post-FEAST surveys; and two email reminders to teachers to complete surveys. The primary investigator (PI) will also assist during teacher training sessions and she or a trained OzHarvest volunteer will be available to assist teachers in data collection, pre- and post-FEAST (subject to COVID19 regulations). Every school that participates in this trial, will be rewarded with a gift voucher, worth $100.00 AUD, if the students complete both pre- and post-FEAST surveys and the teachers complete the program evaluation survey. Additionally, schools will be eligible to win one of five prizes (worth $100.00 AUD) for the best School Cookbook produced. The cookbooks will be judged by the FEAST education and research teams.

OzHarvest has secured funding for the FEAST program from governmental, corporate and philanthropic sponsors, to cover the costs of the program, including teacher training, curriculum package and cooking equipment. This is offered to schools with an Index of Community Socio-Educational Advantage (ICSEA) below the average ICSEA value of 1000 and to schools that are in regional or remote Australia, that are interested in participating in the program. The ICSEA information is published on *MySchool* website [50]. However, depending on the funding offer some schools will need to fund the purchase of the food/ingredients required for the cooking activities or ask for support from local businesses. The estimated cost of the food required for 30 students to undertake the six cooking activities is $300.00 AUD. Schools with an ICSEA above 1000, will need to cover the costs of the entire FEAST program for themselves (estimated to cost $4000.00 AUD).

### Outcomes and survey instrument development

**Fig. 1:**
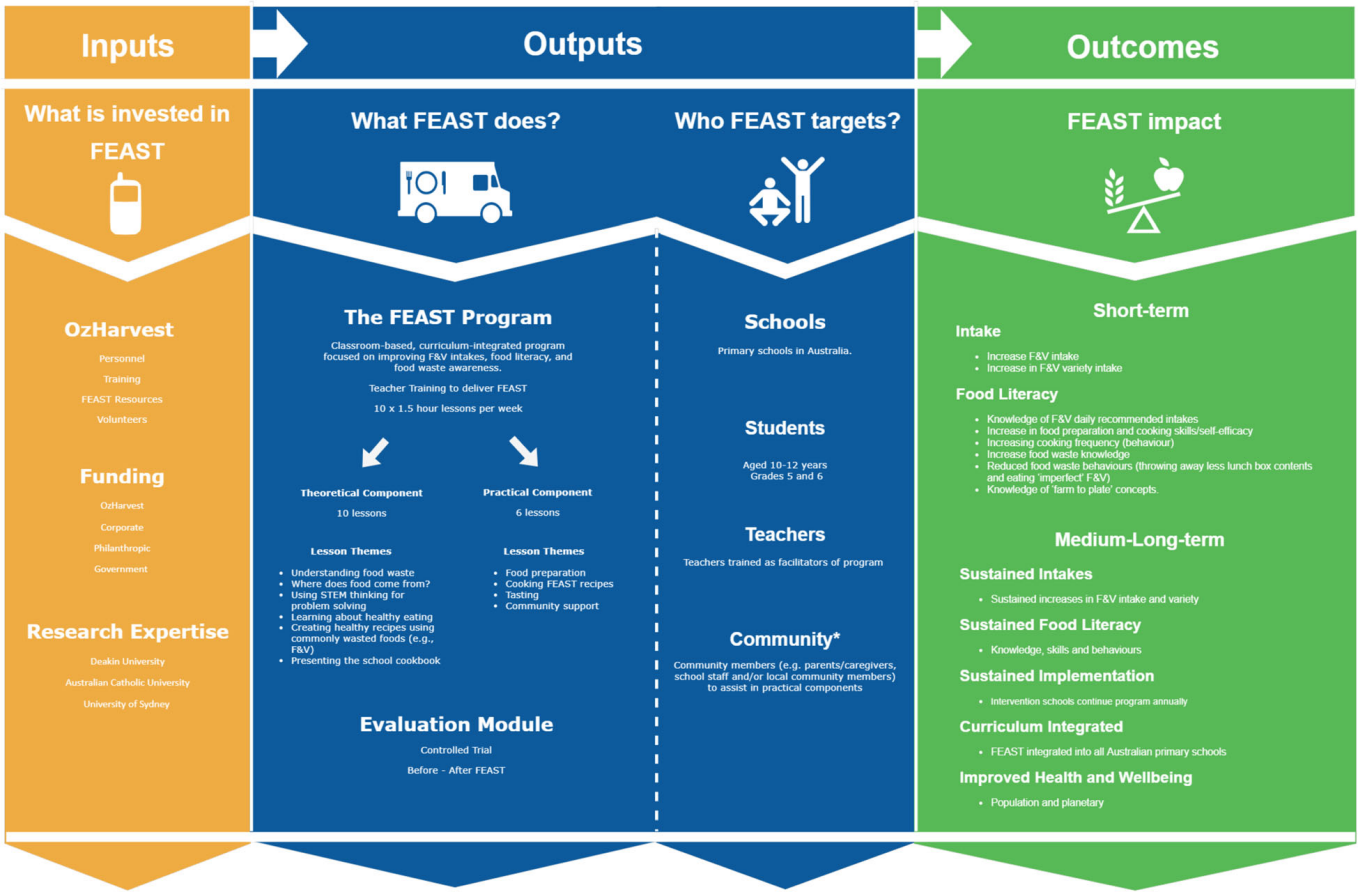
Proposed logic model to guide evaluation of the FEAST program. Legend: F&V fruits and vegetables

**Fig. 2:**
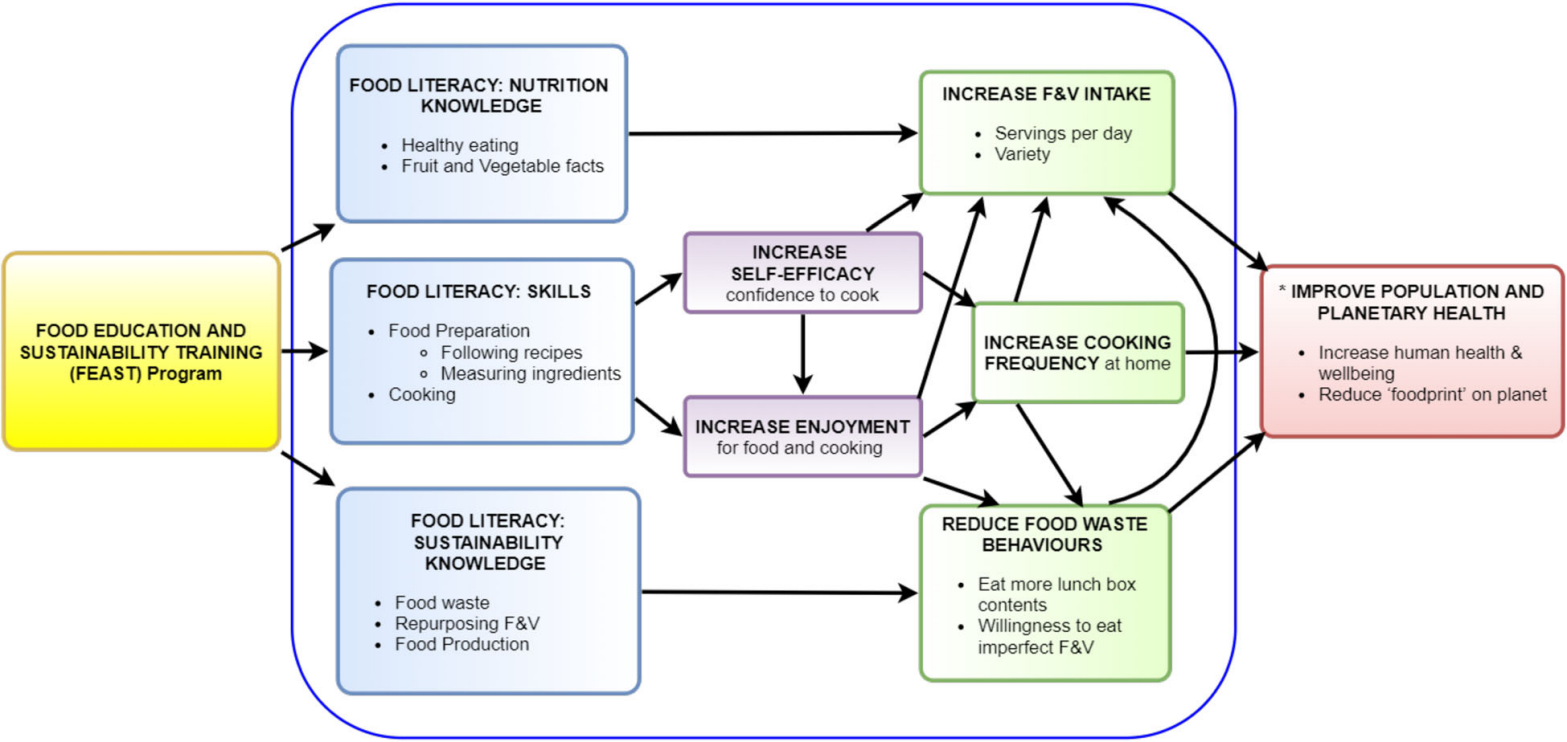
Logic model for hypothesised pathways of effects of FEAST program for student outcomes. Legend: F&V fruit and vegetables; Blue outline denotes short-term outcomes measured in this trial; * Long-term outcomes not measured in this study

The design of the FEAST evaluation was informed by a program logic model and is depicted in Fig. 1 and the logic model for hypothesised pathways (mediators) of the effects of the FEAST program for participant (student) outcomes, is depicted in Fig. 2. The medium- and long-term outcomes will not be assessed, but have been included in both figures for thoroughness.

As there was no survey instrument that captured all of the constructs for this study, a survey tool was developed specifically for this trial. While there are many definitions of food and nutrition literacy [51] the following definition from a scoping review [52] was used to guide survey development, as it encompassed the constructs being taught in the FEAST program, as well as those being examined (plus others, not examined) in this trial:

*“Food literacy is the ability of an individual to understand food in a way that they develop a positive relationship with it, including food skills and practices across the lifespan in order to navigate, engage, and participate within a complex food system. It’s the ability to make decisions to support the achievement of personal health and a sustainable food system considering environmental, social, economic, cultural, and political components.”*

*This definition included knowledge acquisition in the following areas:*

(i) *Knowledge of the effect of food on personal health and wellbeing;*
(ii) *Knowledge of the food system from production to access to waste; and*
(iii) *Knowledge of the broader context of the food system including social, economic, cultural, environmental and political factors”* [52].

Questions were derived from a number of different published children’s nutrition, cooking skills and food waste behaviour surveys/questionnaires. Input into survey development was sought from key stakeholders: the FEAST manager (high school design and technology teacher and registered nutritionist) and FEAST coordinator (high school teacher, > 20 years of experience working in schools in sustainability and STEM); a biostatistician (who is also a behavioural scientist); and experts in child and adolescent nutrition research (i.e. the research team, including nutritionists, behavioural experts and a paediatrician). The survey was piloted with a group of eight, 9–12-year-old boys and girls, to test for ease of reading, comprehension and time to complete. No child reported any issues with comprehension, and completion times ranged between 15 and 25 min.

A 25-item survey has been developed to capture student self-reported measures. The survey starts with asking students for basic demographic information followed by three sections covering: (i) nutrition/intake (six questions); (ii) food preparation/cooking (eight questions); and (iii) food waste/production (11 questions). (Refer to Additional file 5 for full details of all questions and response options for the student surveys.)

#### Primary outcomes

There will be two primary outcome measures: individual fruit (excluding juices) and vegetable (including potatoes and legumes) consumption (measured as serves consumed/day). Changes in consumption of F&V servings/day, will be calculated as the mean difference, between baseline (pre-) and post-intervention (with 95% Confidence Intervals [CI]). A standard serve of fruit is approximately 150 g (approx. 5.3 oz) and a standard serve of vegetables is approximately 75 g (approx. 2.6 oz) [53].

The first two questions, that relate to children’s F&V consumption, have been chosen from the food frequency questionnaire (FFQ) used in the *Many Rivers Diabetes Prevention Project* [54]. This FFQ has been used among Australian children aged 10–12 years, and has been assessed for and found to have, criterion/concurrent/predictive validity and reliability [55].

#### Secondary outcomes

Secondary outcomes will include nutrition consumption and food literacy constructs such as: nutrition knowledge, food preparation and cooking (skills, self-efficacy and behaviour), food waste (knowledge and behaviour), and food production (knowledge). All outcomes will be calculated as differences between baseline (pre-) and post-intervention.

##### Nutrition (F&V consumption, variety and knowledge)

The proportion of children consuming the recommended two serves of fruit/day will be calculated. According to the Australian Bureau of Statistics, only 6.3% of children meet their recommended intake of five vegetables/day, with an average intake of only two serves/day [7]. Therefore, a more conservative level of two serves/day will be used to assess changes in proportion of students consuming vegetables. For analyses involving proportions, intakes of F&V will be dichotomized to 0–1 serves/day verses ≥2 serves/day. These outcomes will be calculated using the same two questions used for the primary outcomes.

Vegetable variety (number of different types of vegetables consumed yesterday), fruit variety (number of different types of fruits consumed yesterday), and the mean number of the variety of the different F&V consumed, will be calculated (with 95% CI), as well as the proportion (in percentages %) of students consuming these F&V. To capture variety of F&V eaten by children, two questions were taken from the *Modified Child Nutrition Questionnaire* (MCNQ) [56], which was designed to assess dietary patterns in Australian children aged 9–13 years, and has face/content validity, criterion/concurrent/predictive validity and reliability [55]. However, there has been an addition of ‘other’ under the list of fruits and vegetables listed in these questions, with the opportunity to state which ‘other’ F&V children consume. This was used in order to capture the different varieties of F&V consumed by children from culturally and linguistically diverse (CALD) backgrounds. The inclusion of questions that are sensitive to populations that are CALD, was one of the lessons learnt from the FEAST pilot study, given the schools that participated in the pilot, had a high percentage of students that were from diverse ethnic backgrounds, as well as a small percentage of students from Indigenous backgrounds.

Nutrition knowledge will be calculated as the proportion (%) of students reporting knowing the recommended intakes of fruit and vegetable serves/day. Questions for this outcome were also taken from the MCNQ [56], however the multiple-choice responses were modified and simplified to include only specific servings/day with the exclusion of responses that included servings/week.

##### Cooking (skills, self-efficacy and behaviour)

The proportion (%) of students reporting the following, will be calculated: the ability to prepare food (i.e. fruit snack, vegetable snack, salad); help family make a meal; measure ingredients; cut food; follow a recipe; and the number of times/week they help their family cook dinner at home. Also, the mean score of the number of ‘yes’ responses to these questions will be calculated out of a possible seven ‘yes’ responses, and compared between baseline and post-intervention. The seven questions to assess these outcomes were taken from the survey used in the *Cooking with Kids* program and have been validity-tested for self-efficacy among children aged 9–11 years [57]. The question that measures behaviour change (i.e. help their family cook dinner at home), was taken from the *Experiential Cooking and Nutrition Education Program*, that was designed to increase cooking self-efficacy and vegetable consumption in children in Grades 3–8 [58] however, it has not been tested for validity and/or reliability.

##### Food waste (knowledge and behaviour)

The proportion (%) of students reporting the amount of their school lunch consumed; why they consumed that much; what they do with the food they do not eat; as well as why and how they dispose the food they do not eat, will be calculated. The four questions for this outcome were modified from one published study investigating food waste behaviours among children in Western Australian upper primary and lower secondary schools (Grades 5–8) [59]. However, this survey has not been validated to date. Most validated measures for school mealtime waste involve observations and assessments of food plate waste, such as digital photography [60, 61]. This is not an option in this study, as Australian primary schools do not have national school lunch programs as they do in the US [60] and UK [62], instead Australian children are more likely to bring their lunch from home [63] or purchase it from the school canteen [64]. Also, a food waste audit was not feasible as a more intensive program of research would be required and is beyond the time and resources available for this trial.

The proportion (%) of students reporting their understanding of the impact of food waste on the environment; whether they eat ‘imperfect’ F&V; and whether they are willing to eat or use ‘imperfect’ bananas in a recipe, will be calculated. Also, two open-ended questions will be asked of the students: what ‘food waste’ means to them and how they think food waste impacts the environment. The questions for these outcomes were specifically developed for the FEAST program and were aligned with food waste lessons.

##### Food production (knowledge)

Understanding of the ‘farm to plate’ concept, will be calculated as the proportion (%) of students reporting the correct sequence of a strawberry’s journey from farm to plate. The question for this outcome was also specifically developed for the FEAST program and was aligned with the food production lesson.

A combination of dichotomous (yes/no) responses, multiple choice options, Likert scale questions and short sentence answers, make up the surveys. Each survey question has been written in simple language and several questions are accompanied with an illustration to enhance understanding, such as F&V serving sizes. In addition, two posters with the images of the F&V that appear in the questions relating to F&V variety, will be provided to the teachers to display them in the classroom, so that students have additional visual aids to assist them with the F&V questions. For ease of use, the survey was created within the electronic data capture system called REDCap, which will entail the self-reported pre- and post-intervention surveys, which should take between 20 and 30 min to complete. Children aged 10–12 years of age, are capable of self-reporting constructs like these [65].

In order to enhance successful completion of the FEAST survey, the following evidence-based recommendations have been included: short completion time [66]; training of teachers to explain questions and monitor children [65]; use of visual prompts [65, 67]; use of technology, such as online surveys [68]; and reduced retention interval, by asking children to recall what they ate in the previous 24 h [69].

#### Process evaluation outcomes

The RE-AIM framework (Reach, Efficacy, Adoption, Implementation and Maintenance) [70] has been adopted to guide the process evaluation along with the inclusion of two other parameters. The evaluation of the ‘efficacy’ component (or in this real-world trial ‘effectiveness’) has been described above, under ‘outcomes’. This process evaluation will assess program: reach (to students and teachers); adoption (by schools); implementation (training of teachers, adherence by students and teachers; barriers and facilitators to implementation); maintenance (intention by students and teachers); satisfaction (of students and teachers); and perceived benefits (by teachers for their students). (Refer to Additional files 6 and 7 for full details of all questions and response options for the student and teacher process evaluation surveys, respectively).

##### Reach of program (teacher and student)

Reach may be defined as the proportion of participants who are eligible but did not participate in some or all of the intervention compared to those who participated in all of the intervention [70]. To evaluate the FEAST program’s reach, the post-intervention teacher survey includes questions that ask teachers: how many students in their class and how many of their students participated in the FEAST program.

##### Adoption of program

Adoption refers to the proportion of settings that adopt a given program [70]. This can be assessed by involving the program creators and educators of the FEAST program (i.e. OzHarvest) and asking them to provide the numbers of schools approached and the number of schools that have adopted the program.

##### Implementation (training of teachers, adherence by students and teachers; barriers and facilitators)

Implementation can be defined as the extent to which a program/intervention was delivered as intended, which can be examined at the individual-level and program-level [70]. To monitor adherence/fidelity at the program-level, and to investigate whether teachers delivered the intervention as intended, the post-intervention teacher survey includes questions that ask teachers: what type of training they undertook (face-to-face vs online vs no training); if they implemented the program alone or with another teacher; how many of their students participated in the FEAST program; how many FEAST lessons were conducted; how many cooking sessions were conducted; and if the class created a school cookbook. To monitor adherence at the individual (student level) there is a question embedded within the student post-intervention survey, asking students whether they participated in the cooking activities.

To evaluate whether teachers received all of the resources required to implement the program, embedded within the question to rate the effectiveness of the resources, teachers will also need to ‘tick’ the resources they did not receive.

To gauge potential barriers to implementation, teachers will be asked: if there were any classroom, school or external barriers that impeded students undertaking the FEAST program; whether cost was a barrier to delivering the program; and whether finding volunteers to help with the practical components was an issue.

To gauge potential facilitators, teachers will be asked open-ended questions such as: to describe their favourite aspect of the program; which aspects of the program they thought had the greatest impact on their students; whether they would continue implementing the program and why/why not. Lastly, they will be asked whether they have any feedback, and if they have any suggestions as to how OzHarvest could modify the program in the future.

##### Maintenance

Maintenance can be defined as the extent to which a program is sustained over time, at both the individual and the organization level [70]. As the evaluation process will take place immediately post-intervention, it will not be possible to measure maintenance over time. However, the intention to maintain the program beyond initial implementation, will be assessed at the teacher and student levels. Teachers will be asked: “*will you continue implementing the FEAST program in your classroom?*” and depending on their response (‘Yes/No’), the appropriate open-ended question will also be asked: “*what motivates/inspires you to continue implementing the FEAST program in your classroom?*” or “*what are the reasons you would not continue to implement the FEAST program in your classroom?*” For the students, they will be asked: “*would you like to do the FEAST program again?”*

##### Program satisfaction (teacher and student)

To assess satisfaction, the teachers will be asked: if they felt the training was effective and whether it helped prepare them to deliver the program within the classroom setting; to rate the effectiveness of the resources provided; whether the program met Grade 5–6 KLAs, aligned with the cross-curricular priority of sustainability and general capabilities and whether it was easy to implement STEM lessons; whether it met their student’s learning needs; and whether it was easy to integrate the program into the classroom routine. Teachers will also be asked whether they thought their students found the activities and website easy to follow and resources engaging.

To assess satisfaction, students will be asked whether: FEAST activities and website were easy to read and understand; if they thought the program was fun; if they enjoyed cooking; if they cooked some of the classroom recipes at home; whether they learnt anything new about food preparation and cooking; and whether they created a cookbook and if so, if they enjoyed creating it. Both student and teacher post-FEAST surveys ask them to rate how likely they would recommend the FEAST program to others.

##### Program perceived benefits (teacher and student)

The teachers will be asked whether they thought their students understood the importance of being aware of food waste; the impact of food waste locally and globally; and whether students understood which behaviours could reduce food waste in the home and at school. Teachers will also be asked whether they thought: students understood where food comes from; were making healthy food choices; understood how to prepare and cook food; were eating more F&V and less junk food; and were reducing food waste behaviours (and by how much i.e. what percentage reduction). There is also one open-ended question asking students to name one new thing they learnt during the cooking activities.

### Participant timeline

**Fig. 3:**
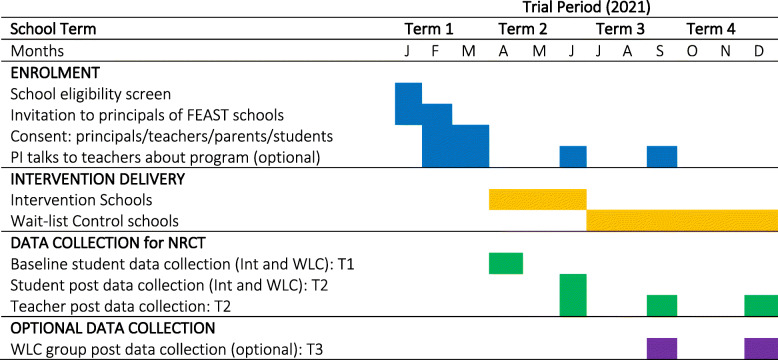
Timeline of school enrolments, intervention delivery and data collection. Legend: PI Primary Investigator (FK); Int Intervention group; WLC Wait-list control group; T1 Timepoint 1; T2 Timepoint 2; T3 Timepoint 3

For the intervention school groups, all baseline (i.e. pre-intervention) student surveys will be issued in week one of the school term in which the FEAST program will be implemented (i.e. Timepoint 1: T1). Post-intervention student surveys will be issued in the last week of the same school term (i.e. Timepoint 2: T2). Figure 3 outlines the trial timeline for school enrolments, intervention delivery and data collection throughout 2021.

### Sample size

Sample size requirements estimated that 20 schools (10 per intervention arm) will be required to take part in this study, with an average of 50 students (SD ± 22, and therefore CV = 0.436) per arm. These numbers are feasible given that more than 30 schools have enrolled to participate in the program with OzHarvest, for 2021, and recruitment continues. A within-school intraclass correlation of 0.04 is estimated for both primary outcomes of fruit and vegetable intakes [71–73] and correlations of 0.5 are assumed between outcome measures between baseline and 10 weeks [56]. Standard deviations for vegetable and fruit serves/day are estimated to be 1.3 and 1.1 respectively, according to the South Australian Health Reports from 2015 on children’s vegetable intake [74] and fruit intake [75]. Allowance will be made for 20% loss due to follow-up or missing data [72, 76].

Given there are two primary outcomes (fruit serves/day and vegetable serves/day), a Bonferroni-corrected alpha (α) of 0.025 (i.e. 0.05/2) will be used. An increase of 0.5 servings of fruit/day (i.e. 75 g) and vegetables/day (i.e. 37.5 g) may be considered meaningful [56, 77], given that even small increases (e.g. 50 g/day) in combined F&V intakes have the potential to provide protection against the development of certain NCDs [77]. Under the above assumptions, the study would provide 90% power to detect an intervention effect of this size, for the vegetable intake primary outcome and 97% power for the fruit intake primary outcome.

### Recruitment

As part of their recruiting strategy, OzHarvest provides avenues via: media and communications (e.g. *media* releases, word of mouth, school TV shows, and developing and maintaining partnerships with external organizations, such as Nutrition Australia and Sustainable Schools NSW); FEAST promotional activities (e.g. school presentations, parental engagement, video assets, and attending sustainability conferences); as well as digital marketing (e.g. email communications with interested schools). Schools that become interested in the program, register their interest to participate via the OzHarvest web portal [78].

Accordingly, the method of recruitment for this trial has two parts: (a) self-selection by the schools to participate in OzHarvest’s FEAST program in 2021; and (b) invitation by the primary investigator (FK) to the principals of enrolled schools, inviting him/her to allow their school to participate in this trial.

### Assignment of interventions

As this is a pragmatic school-based study, implemented by OzHarvest, randomization will not be possible. The schools self-select to undertake the FEAST program during the school term that best fits in with their academic program, and they also choose whether or not to participate in this trial.

### Blinding (masking)

As with many cluster trials, this trial will be pragmatic in nature as it involves participating in a program in a real-world setting (i.e. primary schools in NSW) and as such, blinding of participants (i.e. students and teachers) will not be possible [79]. However, the biostatistician involved in data analyses will be blinded to group assignment.

### Data collection methods

The class teachers participating in the FEAST trial, will receive a URL link to access student surveys, on the REDcap platform, in the last week of the school term preceding implementation. The teacher will then email this link to his/her entire class in week one of the school term, that FEAST will be implemented. At the end of the school term, a second URL link will be issued to the class teachers to access the post-FEAST survey. The teachers will set up a time for all students in their class to complete the pre-FEAST survey prior to starting the program (T1) and the post-FEAST surveys on program completion (T2). Schools acting as the wait-list controls will also complete the surveys at the same time as intervention schools (i.e. at T1 and T2). The PI (FK) and/or OzHarvest volunteers will assist the teachers in data collection (subject to COVID19 regulations).

### Data management

Data will be stored securely as per the requirements of the Deakin University, Human Ethics Advisory Groups for the Faculty of Health. Data will only be accessible to the research team and trial biostatistician. Confidential participant (student and teacher) and school data, will be deidentified and stored securely and will not be linked to survey responses.

### Statistical analysis

Demographic and baseline characteristics will be summarised for both intervention and wait-list control groups and compared for differences at baseline. Standard summary statistics (mean and standard deviation [SD]) or non-parametric statistics (medians and inter-quartile ranges) where applicable, will be used. For the categorical variables, frequencies and percentages will be calculated and reported.

Effects of the intervention on study outcomes will be estimated with generalized linear mixed models (GLMMs) including random intercepts to account for clustering within schools and fixed effects of intervention arm, using appropriate family and link functions according to outcome distribution or type. Models will be adjusted for potential confounders: gender, grade, ICSEA, type of teacher training (face-to-face vs online vs no training), and whether the student speaks another language at home (which will be dichotomized as Yes/No). Analyses will also adjust for baseline levels of the desired outcome. Intracluster correlation coefficients (ICC) for within-school clustering in each intervention effect model will be calculated and reported. Similarly, within-group changes in outcomes will be estimated using GLMMs including random effects for schools and individuals, and fixed effects of time. Estimated intervention effects and within-group changes will be reported as unstandardized regression coefficients or exponentiated coefficients (e.g. odds ratios), with 95% confidence intervals and *p*-values.

All analyses will follow the intention-to-treat (ITT) principles and missing data will be handled using multiple imputation by chained equations [80]. Imputation of outcome variables (and covariates if missing) will be conducted separately by intervention group and will include schools and covariates as auxiliary variables [80].

To assess sensitivity of the findings to different assumptions around the missing data mechanism, complete case analyses (valid under a missing completely at random assumption) will also be conducted as a sensitivity analysis. No interim analyses of trial outcomes are planned nor any stopping rules.

Analyses for the process evaluation, involving responses from students and teachers, will involve descriptive statistics for the quantitative component using standard summary statistics. While the qualitative component, which involves responses to open-ended questions, will be analysed using both content and thematic analyses, as well as qualitative description [81, 82].

### Harms

As this is an evaluation of the FEAST program, it is not anticipated to produce adverse reactions/events. To date, no issues have been reported from the 170 schools that have implemented FEAST between 2018 and 2020. To our knowledge, there are no known published studies in school-based nutrition experiential interventions reporting adverse reactions/events. However, this study has included four questions in the teacher evaluation to assess ‘harms’. The survey includes questions that ask teachers whether: they completed the mandatory FEAST Program risk assessment prior to delivering the program to their students (Refer to Additional file 4 for description of risk assessment components); whether any students were harmed; if they were harmed, how many were harmed and what types of harms occurred in the classroom setting over the course of the FEAST Program. (Refer to Additional file 7 for full details of questions and response options on harms in the teacher surveys.)

## Discussion

The implementation of the FEAST program by OzHarvest, across primary schools in NSW, provides an invaluable opportunity to gain scholarly and translational research outcomes. As such, this paper has described the protocol for a pragmatic, non-randomized controlled trial involving a wait-list control group. The evaluation of FEAST will contribute to a growing body of work investigating the effectiveness of school-based interventions incorporating nutrition and sustainability education with experiential activities.

There are both strengths and limitations to the evaluation design. It has been acknowledged that using a non-randomized design has the potential to introduce selection bias [79]. However, due to the pragmatic nature of this study, which will be conducted in the real world, where schools will self-select to participate in both the FEAST program and subsequently in the trial, randomization will not be possible. Despite this potential for bias, using a wait-list control with pre- and post-measures, should help mitigate some of the issues related to this type of bias. Other protections from potential bias when using a NRCT design include the provisions of a detailed protocol with pre-specified statistical analysis plan which, should include, predefined primary and secondary outcomes, their derivations from measured variables, methods for managing missing data, as well as planned subgroup and sensitivity analyses [83], all of which, have been included in this protocol description.

Another limitation of this study is the reliance on self-reported measures from children aged 10–12 years. However, the majority of questions utilised to create the FEAST survey instrument, were taken from reliable and validated surveys that have been tested among this age group [54, 56, 57, 65]. Furthermore, it has been found that cognitive abilities required to self-report food intake increase among children aged 8 years and over [84], and the FEAST program is designed for children over this age. Another concern with self-reported measures, is that there is the possibility that students may answer in a socially desirable manner and as a result they may be over- or underestimating their responses, depending on recall bias. Although this is a common challenge with this type of research, to limit the potential for social desirability bias, the students will be advised prior to survey completion that the survey is not a test, there are no ‘right’ or ‘wrong’ answers, and that it is important for them to report as honestly as possible [85].

Strengths of the FEAST program include the incorporation of a number of components that have shown to contribute to effective school-based nutrition education programs for children, such as the implementation of: multi-component strategies (involving teachers, parents/caregivers and community) [86]; age-appropriate, hands-on experiential activities (such as food preparation and cooking) [86]; exposure to F&V [86]; strategies to enhance fidelity by training the implementers with standardized protocols (i.e. accredited teacher training) [86]; integration of such programs within the curriculum (aligned with the Grades 5–6 key learning areas in the Australian Curriculum) [19]; as well as proper alignment between the objectives, intervention, and desired outcomes [86].

The strengths of the evaluation design include the use of: the 33-item SPIRIT checklist to guide the design of the protocol; using a controlled design; questions for the primary outcomes derived from reliable and validated measures tested on this age group; many of the secondary outcomes, were also derived from reliable and validated measures. The trial is well powered to detect changes in outcomes and the trial statistician will be blinded to group allocation when conducting the statistical analyses. Also, this trial has included qualitative questions embedded within the quantitative surveys. The combination of qualitative and quantitative components within the surveys, will enable the trial’s conclusions to be expanded upon [87, 88]. If triangulation of results is found between qualitative and quantitative components, then convergence of outcomes allows for stronger inferences about findings [87, 88].

Furthermore, non-randomized evaluation designs can contribute data on the effectiveness of interventions, and if conducted and reported systematically, have the capacity to contribute to building evidence-based public health practices [89]. Accordingly, the TREND (Transparent Reporting of Evaluations with Nonrandomized Designs) [89] and the CONSORT (Consolidated Standards of Reporting Trials) statement for pragmatic trials [90] will be used to report outcomes.

Given the challenges of promoting healthy diets, sustainable eating and reducing food waste at the population level, the FEAST program is well-positioned to play a key role in this school-based approach to engage students to eat healthily and sustainably. Initiatives undertaken within the school system have the added advantage of reaching large, diverse populations, placing them in a unique situation to deliver universal and equitable public health strategies [11, 23, 33].

If this program demonstrates effectiveness, FEAST will have the potential to benefit students by providing them with a set of tools to help them achieve healthy and sustainable eating practices. FEAST also has the potential to support the Australian Curriculum with health promoting and sustainability messages, which could contribute to: health promotion within schools [91]; sustainable schools initiatives [92]; government-supported public health initiatives [4, 93–96]; prevention of chronic conditions, in the long term [97, 98]; the national agenda to reduce food waste [36] as well as to the sustainable development goal targets for 2030 [92, 99, 100].

## Supplementary Information

**Additional file 1.** Comparison between the components of FEAST and other sustainable food initiatives

**Additional file 2.** Resources for the FEAST Program

**Additional file 3.** FEAST Program Lesson Plan Topics for Teachers

**Additional file 4.** FEAST Program Practical Guide for Teachers

**Additional file 5.** FEAST Student Survey

**Additional file 6.** FEAST Student Evaluation

**Additional file 7.** FEAST Teacher evaluation

## Data Availability

Data not applicable for a protocol. Other than information published in this protocol, FEAST training materials remain the property of OzHarvest.

## Abbreviations

NCDs: Non-Communicable Diseases
F&V: Fruits and Vegetables
FEAST: *Food Education and Sustainability Training*
PPM: Precede-Proceed Planning Model
SCT: Social Cognitive Theory
NSW: New South Wales
SPIRIT: Standard Protocol Items for Clinical Trials
NRCT: Non-randomized Controlled Trial
KLAs: Key Learning Areas
STEM: Science, Technology, Engineering, and Mathematics
PI: Primary Investigator
ICSEA: Index of Community Socio-Educational Advantage
CI: Confidence Intervals
T1: Timepoint 1
T2: Timepoint 2
T3: Timepoint 3
SD: Standard Deviation
FFQ: Food Frequency Questionnaire
MCNQ: Modified Child Nutrition Questionnaire
CALD: Culturally and Linguistically Diverse
GLMMs: Generalised Linear Mixed Models
ICC: Intracluster correlation coefficients
ITT: Intention-To-Treat Analysis
TREND: Transparent Reporting of Evaluations with Nonrandomized Designs
CONSORT: Consolidated Standards of Reporting Trials
