# Supplementary material for: Evaluating OzHarvest’s primary-school Food Education and Sustainability Training (FEAST) program in 10–12-year-old children in Australia: protocol for a pragmatic cluster non-randomized controlled trial
Source: BMC Public Health. 2021 May 22;21:967. doi: 10.1186/s12889-021-10302-0 (PMC8140478; doi:10.1186/s12889-021-10302-0)
Supplement: Supplementary file 1 — Additional file 1. Comparison between the components of FEAST and other sustainable food initiatives [file 12889_2021_10302_MOESM1_ESM.pdf]

Program name (Country)	School-Based	Curriculum-integrated	Nutrition Education	Sustainability Education	Experiential Activities	Food Systems/ Production	Food Waste	Community Involvement
Food Education and Sustainability Training (FEAST) (Australia)	✓	✓	✓	✓	✓	✓	✓	✓
Farm-to-School (US) [35]	✓		✓*		✓	✓	✓*	✓
The Healthy Planet, Healthy Youth (US) [29]	✓	✓		✓		✓	✓	
Food for Life (UK)[33]	✓	✓		✓	✓	✓		✓
Farm to Cafeteria (Canada)[31]	✓	✓	✓		✓	✓		✓

Legend: Void implies the program did not include that component; * Optional component
