# Supplementary material for: Evaluating OzHarvest’s primary-school Food Education and Sustainability Training (FEAST) program in 10–12-year-old children in Australia: protocol for a pragmatic cluster non-randomized controlled trial
Source: BMC Public Health. 2021 May 22;21:967. doi: 10.1186/s12889-021-10302-0 (PMC8140478; doi:10.1186/s12889-021-10302-0)
Supplement: Supplementary file 2 — Additional file 2. Resources for the FEAST Program [file 12889_2021_10302_MOESM2_ESM.pdf]

Resources	Explanation
Professional Learning Program	The FEAST Professional Development teacher training informs and educates teachers to deliver the Grade 5 - 6 program. The module embraces the latest interactive teaching and learning approach; Solution Fluency and provides teachers with instructional videos and practical activities to be implemented in the classroom.<br><br>OzHarvest offers a NSW Education Standards Authority (NESA) accredited 6 hour face-to-face training at the OzHarvest offices. Also on offer is a 3-hour NESA accredited online training module that is flexible, affordable and practical.<br><br>https://feast.ozharvest.org/event/feast-teacher-training-online
Caregiver and Volunteer Training	The FEAST volunteer training is available via the online platform Go1. The 2-hour training course ensure all volunteers are confident to assist teachers in the FEAST practical classes and that they have the food safety and hygiene and NSW Health COVID-19 training required.
FEAST Classroom Curriculum Package	THEORY LESSON RESOURCES:<br><br>**Unit of Work** - everything required to teach the program including: teacher notes, information about the teaching and learning approach, fact sheets, and curriculum descriptors.<br><br>**STEM Lesson Plans -** a 10-week guide to help deliver the practical and theory components of the program. Includes **OzHarvest video resources library, such as:** video describing the charity's fight on food waste.<br><br>**Teacher Check List** - handy checklist to make sure you are prepared to deliver FEAST in your school.<br><br>**Student Resources -** worksheets for students, including a guide for the creation of a School Cookbook (for the creation of recipes that use foods that are otherwise wasted for e.g. F&Vs).<br><br>**Teacher Answer Booklet -** providing detailed responses to the Student Resources.<br><br>**FEAST Optional Learning Experiences -** such as - how to promote the School Family Cookbook with a school community book launch, and other sustainable ways to reduce one's 'foodprint'.<br><br>**FEAST Recipe Template**- The FEAST recipe template can be used in the School Cookbook.<br><br>PRACTICAL LESSON RESOURCES:<br><br>**Student Role Template-** Let your Healthy Helpers change roles at every cooking session and use the Student Role Template to keep track.

	Hand Washing Poster- Use this poster to teach your students how to wash their hands in a fun and engaging way!<br><br>FEAST Practical Guide - The Practical Guide includes a detailed Risk Assessment and practical checklists to ensure your cooking classes run smoothly and safely (Refer to Table 3).<br><br>Cold Recipe Booklet - six FEAST Cold Recipes designed to provide a fun hands-on learning experience.<br><br>Hot Recipe Booklet - six FEAST Hot Recipes designed to provide a fun hands-on learning experience, while working with heat.<br><br>ONLINE LEARNING – IN RESPONSE TO COVID-19:<br><br>Teacher Instructions - learn how to best use these online learning resources and adapt to your students' needs.<br><br>8 Interactive Lessons - to allow students to complete the theory aspects of the program at home.<br><br>Practical Learning - download the FEAST Cooking at home considerations to start cooking at home.<br><br>School FEAST awards- Sent via email to teachers.<br><br>Support - Online individual support from OzHarvest.<br><br>https://feast.ozharvest.org/shop/viewitem/feast-classroom-curriculum-package
Class Kitchen Kit and Apron pack	(i) The FEAST kitchen kit provides enough cooking equipment for a class of 30 students to become immersed in hands-on cooking activities in the classroom. (No kitchen required)<br><br>Classroom Kitchen Kit Includes:<br><br>- Strainers x 3<br>- Chopping Boards x 12<br>- Mixing Bowls x 9<br>- Slotted Turners x 6<br>- Tea Towels x 12<br>- Measuring Cups (Set of 4) x 3<br>- Measuring Spoons (Set of 4) x 3<br>- Table Forks x 12<br>- Can Opener x 1<br>- Rolling Pins x 6<br>- Box Grater x 3<br>- Child Friendly Knives x 12<br>- Tongs x 6<br>- Mixing Spoons x 6

	https://feast.ozharvest.org/shop/viewitem/feast-kitchen-kit<br><br>(ii) 30 OzHarvest aprons for students to wear during cooking activities in the classroom. (Optional)<br><br>https://feast.ozharvest.org/shop/viewitem/ozharvest-apron-pack--450--gst-inclusive-
Electric Frying Pans	6 Electric frying pans per class, so students can make the six hot recipes.
FEAST at Home	Activities and recipes for parents/caregivers to start cooking with their children at home.<br>Activities to reduce food waste at home.<br>Activities and games for the family to engage in, about healthy eating and food waste.<br><br>https://education.ozharvest.org/home-resources/
