# Supplementary material for: Evaluating OzHarvest’s primary-school Food Education and Sustainability Training (FEAST) program in 10–12-year-old children in Australia: protocol for a pragmatic cluster non-randomized controlled trial
Source: BMC Public Health. 2021 May 22;21:967. doi: 10.1186/s12889-021-10302-0 (PMC8140478; doi:10.1186/s12889-021-10302-0)
Supplement: Supplementary file 3 — Additional file 3. FEAST Program Lesson Plan Topics for Teachers [file 12889_2021_10302_MOESM3_ESM.pdf]

Lesson No.	Title	Activities (Theory and Practical)
Lesson 1	The Essential Question and Scenario	Introduce the unit essential question, scenario and student task
Lesson 2	Understanding food waste	Knowledge - local food waste.<br>Behaviours – how to reduce food waste.<br>Food production – ‘farm to plate’ concept.<br>Cooking activity: Peach parfait
Lesson 3-	Change-makers and where does food come from?-	Change Makers:<br>OzHarvest and Ronni Kahn - What has she changed?<br>Where does food come from?<br>Cooking activity: Fruit Skewers with Natural Yoghurt or Banana Pikelets
Lesson 4-	Using STEM Thinking for Problem Solving-	STEM thinking and science inquiry skills to be change-makers.<br>Creating a hypothesis.<br>Fun Food Science.<br>Creating a Wasty Recipe.<br>Cooking activity: Tzatziki Dip with Vegetable Sticks or Fast Fritters
Lesson 5-	Understanding food waste-	Facts on Food Waste.<br>Food waste report.<br>Fridge and fruit bowl audit.<br>Cooking activity: Bircher Muesli with Apples and Bananas or French Toast
Lesson 6-	Learning about healthy eating-	What does healthy food mean to you?<br>What are the different food groups?<br>What Food Groups do you eat?<br>Cooking activity: Salad Roll or Rainbow Honey Soy Noodle Stir Fry
Lesson 7	Generating Ideas	Designing a recipe.<br>Cooking activity: Crunchy Noodle Salad or Tortilla Wraps with Butter Bean Hummus
Lesson 8	Finalising the recipe	Designing FEAST Recipe.<br>Drafting the recipe.<br>Final recipe write up.
Lesson 9	Recipe Presentation	Develop presentation using PowerPoint, Key Note or Google Slides.<br>Title slide.<br>Key Food Waste Facts.<br>Introduction.<br>Present recipe and why it is healthy and how it reduces food waste.<br>Present FEAST recipe
Lesson 10	Debrief	Students reflect on their learning and all aspects involved researching and designing recipes
