# Supplementary material for: Evaluating OzHarvest’s primary-school Food Education and Sustainability Training (FEAST) program in 10–12-year-old children in Australia: protocol for a pragmatic cluster non-randomized controlled trial
Source: BMC Public Health. 2021 May 22;21:967. doi: 10.1186/s12889-021-10302-0 (PMC8140478; doi:10.1186/s12889-021-10302-0)
Supplement: Supplementary file 4 — Additional file 4. FEAST Program Practical Guide for Teachers [file 12889_2021_10302_MOESM4_ESM.pdf]

Practical Guide Components	Descriptions and examples (e.g.)
Risk assessment checklist	Checking allergies, intolerances or dietary requirements e.g. nut allergies<br>Hygiene and food handling e.g. hand washing<br>Food safety storage and contamination e.g. clean containers<br>Hot water e.g. scolds<br>Kitchen knives e.g. cuts<br>Graters e.g. grating skin<br>Electric frying pans e.g. burns<br>Trip hazards e.g. frying pan power cord<br>Lifting Injuries e.g. kitchen kit boxes<br>Chemical hazards e.g. detergents<br>Fire/Emergency e.g. fire blanket, extinguisher, exit plan<br>Students e.g. challenging behaviours
Food cost saving tips	Shop at local supermarkets and buy fruits and vegetables from the ‘odd bunch’.
Food waste reduction tips	Buy only what you need, shop with a list, freeze leftover food.
Food orders	FEAST practical cold and hot recipe food order list.
Preparation	List of procedures for teachers for: before/during/after sessions.
