# Supplementary material for: Evaluating OzHarvest’s primary-school Food Education and Sustainability Training (FEAST) program in 10–12-year-old children in Australia: protocol for a pragmatic cluster non-randomized controlled trial
Source: BMC Public Health. 2021 May 22;21:967. doi: 10.1186/s12889-021-10302-0 (PMC8140478; doi:10.1186/s12889-021-10302-0)

FEAST Kids

Welcome to the FEAST survey!

Thank you for participating in the FEAST program.
On the following pages, there are some questions we would like to ask you, if you do not understand a question, please ask for help from the classroom teacher.
This survey asks about what you eat and your school lunches.
Your individual information will be kept private and confidential.

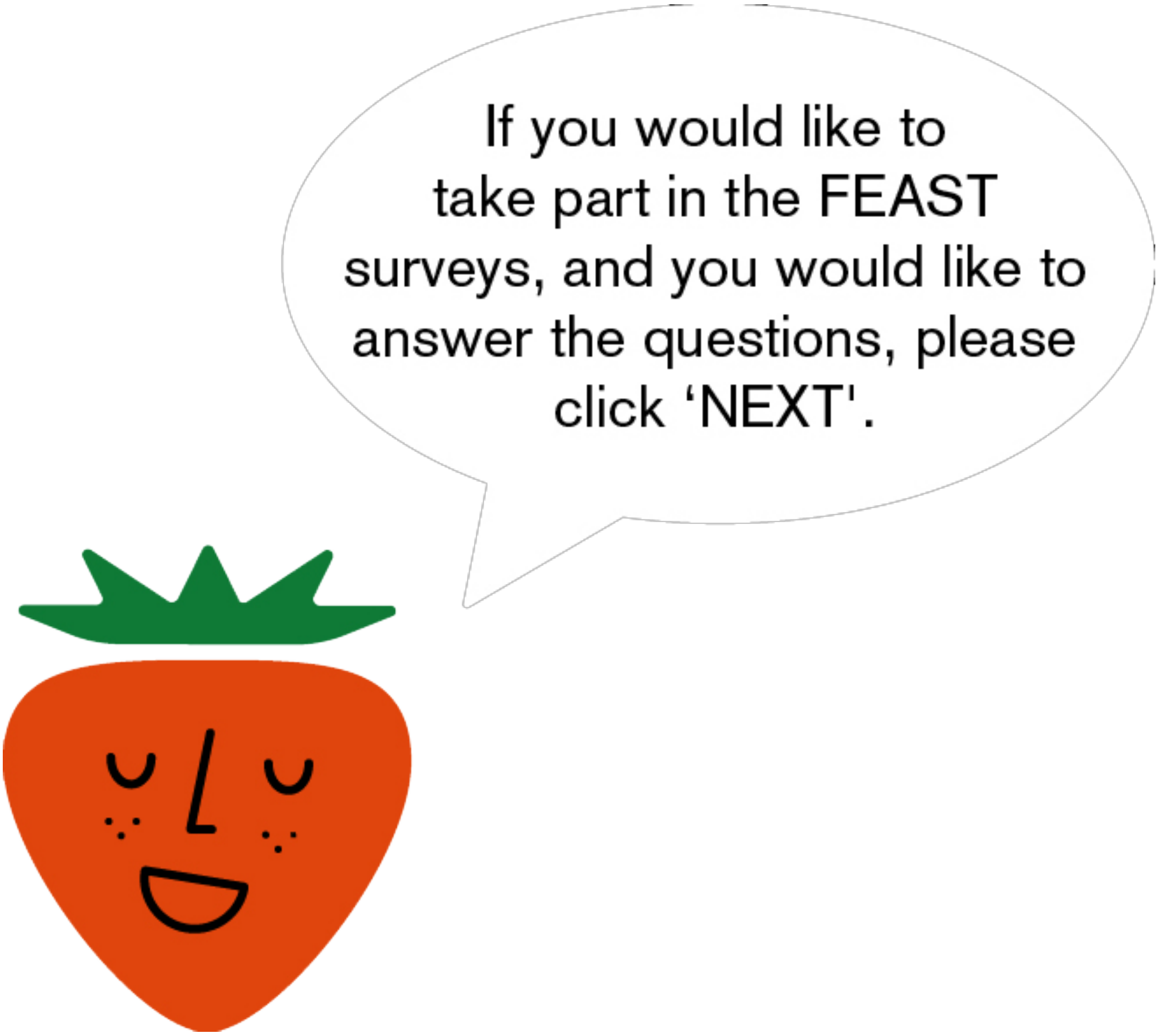

Your School: ______________________

Background

First Name

____

Surname

____

Your school email address:

____

Please enter your school email address again:

____

OOPS! The email address in the two boxes are not the same!
Please make sure that you type your email address correctly in both boxes.

What grade are you in?

◯ Grade 5
◯ Grade 6
◯ Other

What grade are you in?

____

How old are you?

◯ 9
◯ 10
◯ 11
◯ 12
◯ Other

How old are you?

____

What is your gender?

◯ Boy
◯ Girl
◯ Prefer not to say

If you speak another language at home, other than English, please tell us which language it is.

____

Nutrition

Think about what you ate yesterday.
How many serves of fruit do you USUALLY eat, each day?

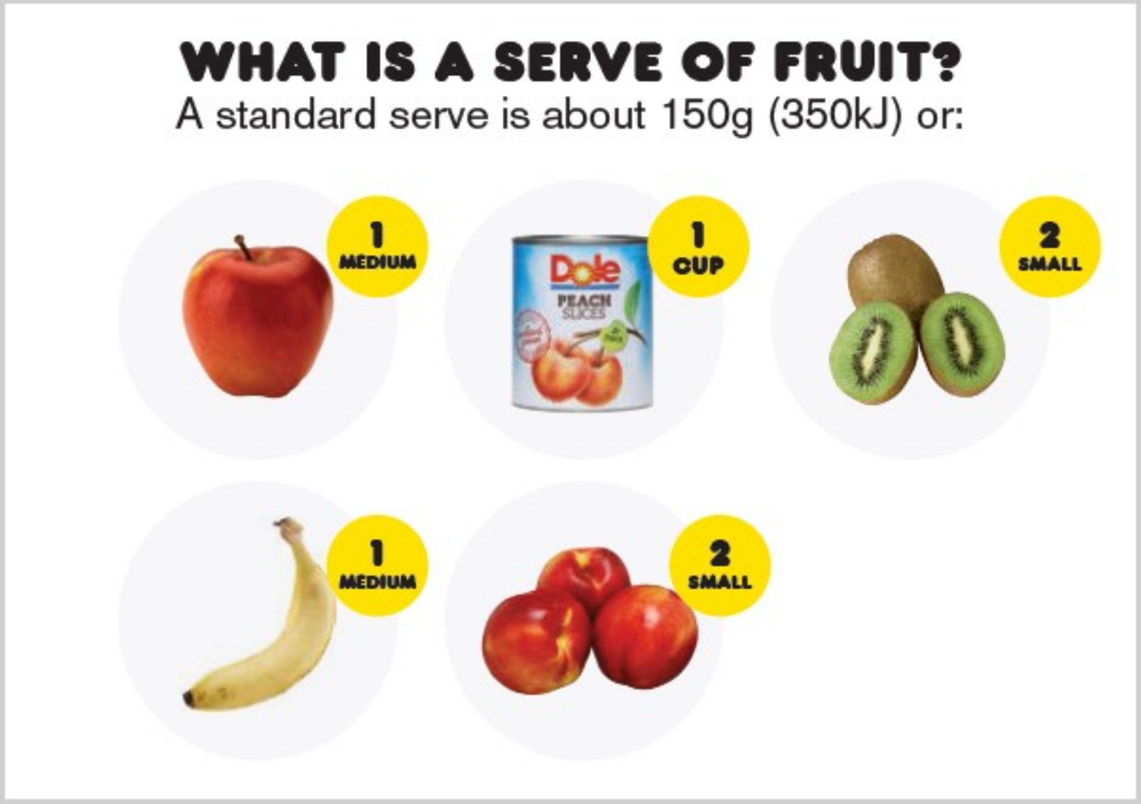

◯ I don't eat fruit
◯ 1 serve or less
◯ 2 serves
◯ 3 serves
◯ 4 serves or more

Think about what you ate yesterday.
How many serves of vegetables do you USUALLY eat, each day?

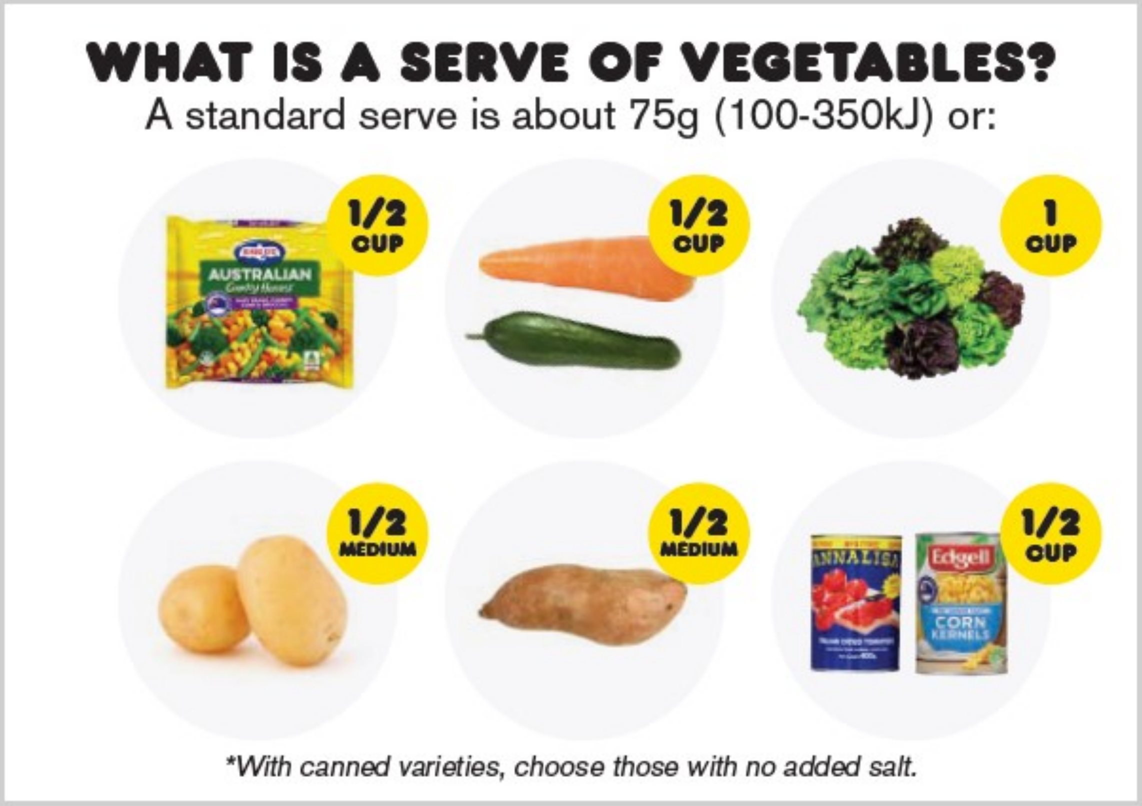

◯ I don't eat vegetables
◯ 1 serve or less
◯ 2 serves
◯ 3 serves
◯ 4 serves
◯ 5 serves or more

Please indicate if you ate this fruit yesterday, by ticking the box or boxes that apply to you.

☐ Apple
☐ Apricot
☐ Banana
☐ Grapes
☐ Kiwi fruit
☐ Mandarin
☐ Nectarine
☐ Orange
☐ Peach
☐ Pineapple
☐ Plum
☐ Rockmelon
☐ Strawberries
☐ Watermelon
☐ Other

What other types of fruit do you eat?

____

Please indicate if you ate this vegetable yesterday, by ticking the box or boxes that apply to you.

☐ Beans (green)
☐ Beetroot
☐ Broccoli
☐ Brussel sprouts
☐ Cabbage
☐ Capsicum
☐ Carrot
☐ Cauliflower
☐ Celery
☐ Chinese greens
☐ Corn
☐ Cucumber
☐ Eggplant
☐ Legumes (baked beans, chickpeas, lentils, kidney beans)
☐ Lettuce
☐ Mushroom
☐ Peas
☐ Potato (not hot chips)
☐ Potato fried eg hot chips/ french fries/wedges
☐ Pumpkin
☐ Spinach
☐ Sweet potato
☐ Tomato
☐ Zucchini
☐ Squash
☐ Other

What other types of vegetables do you eat?

____

How many serves of fruit SHOULD you eat every day?

◯ I don't know
◯ 1 serve
◯ 2 serves
◯ 3 serves
◯ 4 serves or more

How many serves of vegetables SHOULD you eat every day?

◯ I don't know
◯ 1 serve
◯ 2 serves
◯ 3 serves
◯ 4 serves
◯ 5 serves or more

Cooking Skills

	Yes	No	Unsure
I can make a snack with fruit.	◯	◯	◯
I can make a snack with vegetables.	◯	◯	◯
I can help my family make a meal.	◯	◯	◯
I can make a salad.	◯	◯	◯
I can cut up food.	◯	◯	◯
I can measure ingredients.	◯	◯	◯
I can follow recipe directions.	◯	◯	◯

How often do you help cook dinner at home?

◯ Never
◯ Once in a while
◯ A few times a week
◯ Every night or almost every night

Food Waste

On a normal day, how much of your lunch do you eat at school?

◯ Only a little
◯ About half of it
◯ Most of it
◯ All of it

Why do you only eat that much?

Click on as many answers that apply to you.

☐ There is too much food for me to eat
☐ I do not like it
☐ I want to go out and play
☐ I don't have enough time to finish it
☐ Other reasons

What other reasons are there for you not finishing all your lunch?

On a normal day, what do you do with the food that you don’t eat at school?

◯ Throw out at school
◯ Take back home
◯ Throw out somewhere else (like on the way home)
◯ Eat it on the way home
◯ Give it to my friends
◯ Put it in compost bin
◯ I always eat all of my recess and lunch

Why do you throw it out at school?

Click on as many answers that apply to you.

☐ Teacher told me to
☐ Parents told me to
☐ I don't want my parents to see I have left overs
☐ That's what my friends do
☐ That's what my brothers and/or sisters do
☐ That's what I see other students doing
☐ I don't like leftover food in my bag

Why do you take it back home?

Click on as many answers that apply to you.

☐ Parents told me to
☐ Teacher told me to
☐ That's what my friends do
☐ That's what my brothers and/or sisters do
☐ That's what I see other students doing
☐ So that I can eat it at home
☐ So that I don't waste it

Why do you throw it out somewhere else?

Click on as many answers that apply to you.

☐ Teacher told me to
☐ Parents told me to
☐ I don't want my parents to see I have left overs
☐ That's what my friends do
☐ That's what my brothers and/or sisters do
☐ That's what I see other students doing

Why do you throw it in the composte bin?

Click on as many answers that apply to you.

☐ Teacher told me to
☐ Parents told me to
☐ That's what my friends do
☐ That's what my brothers and/or sisters do
☐ That's what I see other students doing
☐ It's good for the planet/earth

What does 'food waste' mean to you?

Does the food that is thrown away, affect the environment?

◯ Yes
◯ No
◯ Unsure

How do you think food waste affects the environment?

What is the correct order a strawberry travels to get to your plate?

◯ farm, home, transport, supermarket
◯ transport, farm, supermarket, home
◯ supermarket, farm, home, transport
◯ farm, transport, supermarket, home

Do you eat blemished or imperfect fruits and vegetables?

◯ Yes
◯ No

Which bananas would you eat or use in a recipe?

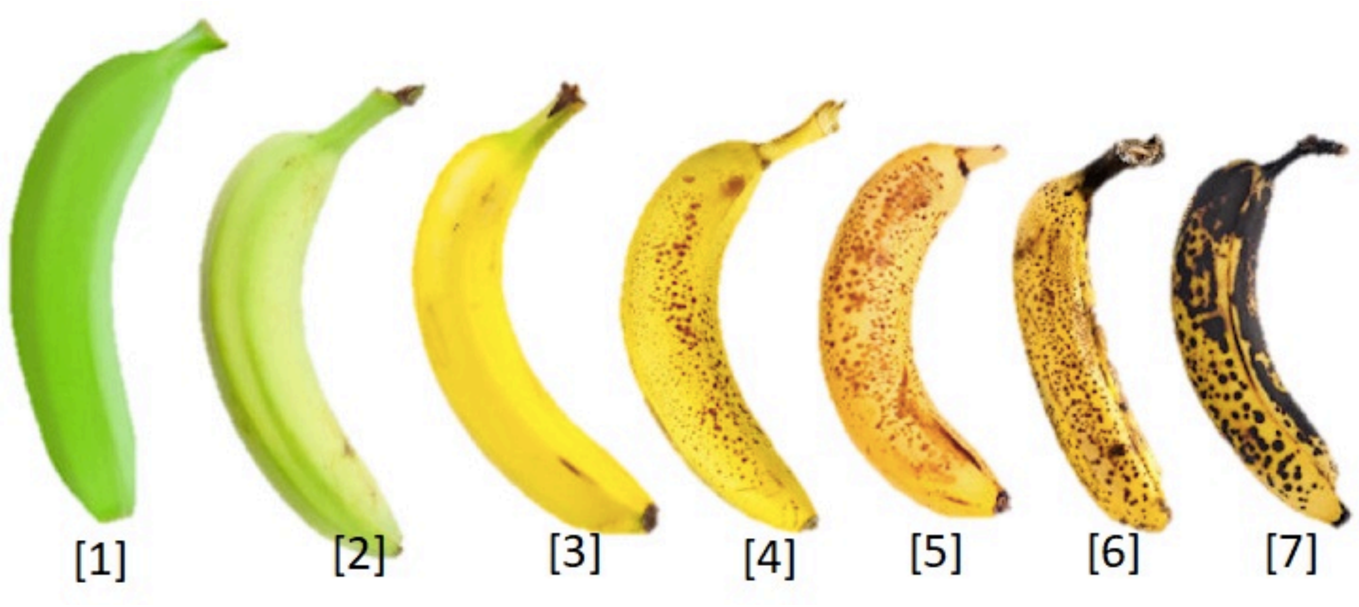

Click any banana(s) that you would eat or use in a recipe?

☐ None of them
☐ [1]
☐ [2]
☐ [3]
☐ [4]
☐ [5]
☐ [6]
☐ [7]

Please remember to click on the "NEXT" button!

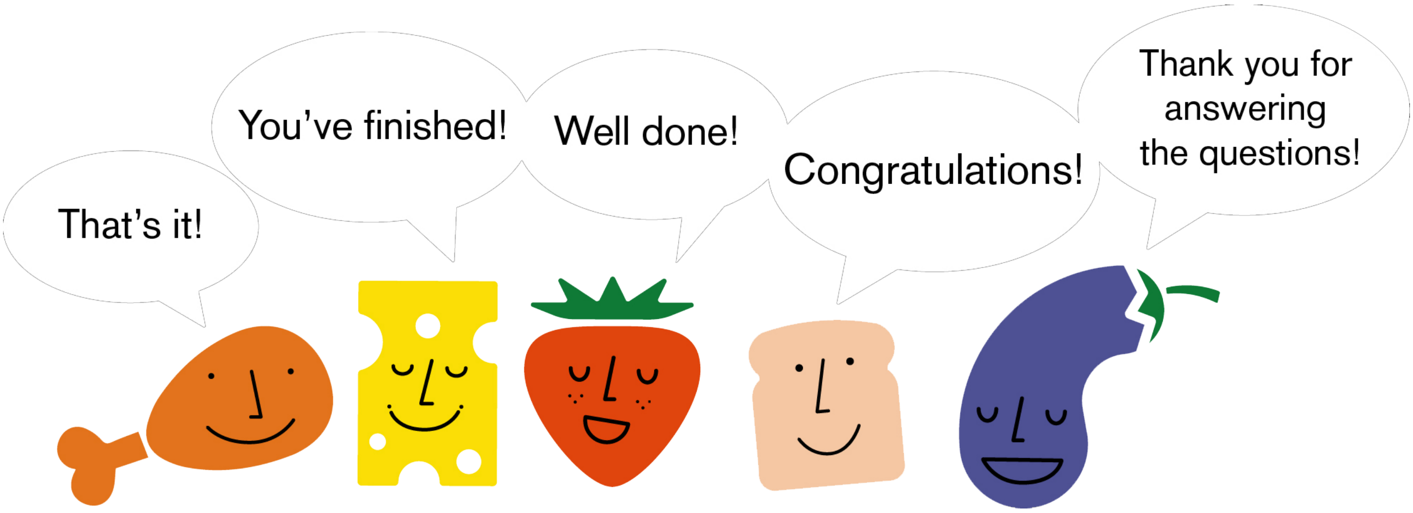

Supplement: Supplementary file 5 — Additional file 5. FEAST Student Survey [file 12889_2021_10302_MOESM5_ESM.pdf]
