# Supplementary material for: Evaluating OzHarvest’s primary-school Food Education and Sustainability Training (FEAST) program in 10–12-year-old children in Australia: protocol for a pragmatic cluster non-randomized controlled trial
Source: BMC Public Health. 2021 May 22;21:967. doi: 10.1186/s12889-021-10302-0 (PMC8140478; doi:10.1186/s12889-021-10302-0)

FEAST Evaluation

What do you think about FEAST?

	Yes	No	Unsure
Were the FEAST activities easy to read?	◯	◯	◯
Were the FEAST activities easy to understand?	◯	◯	◯
Was the FEAST website easy to use?	◯	◯	◯
Was the FEAST program fun?	◯	◯	◯

Did you participate in the cooking activities?

◯ Yes
◯ No

Did you enjoy cooking?

◯ Yes
◯ No

Did you cook some of the classroom recipes at home?

◯ Yes
◯ No

Did you learn something about food preparation and cooking that you did not know before?

◯ Yes
◯ No

Name one new thing you learnt to do during the FEAST cooking activities.

Which was your favourite recipe?

Choose as many as you like.

- ☐ All of them
- ☐ Fruit skewers
- ☐ Tzatziki dip with vegetable sticks
- ☐ Bircher muesli
- ☐ Rainbow salad roll
- ☐ Peach parfait
- ☐ Fast fritters
- ☐ Crunchy Noodle Salad
- ☐ French Toast
- ☐ Banana Pikelets
- ☐ Tortilla Wraps
- ☐ None of them.

Did your class create a cookbook?

- ◯ Yes
- ◯ No

Did you enjoy creating the cookbook?

- ◯ Yes
- ◯ No

On a scale of 0 to 10, how likely are you to recommend the FEAST program to others?

- ◯ 10, Extremely likely to Recommend
- ◯ 9
- ◯ 8
- ◯ 7
- ◯ 6
- ◯ 5
- ◯ 4
- ◯ 3
- ◯ 2
- ◯ 1
- ◯ 0, Not likely to Recommend

Would you like to do the FEAST program again?

- ◯ Yes
- ◯ No

Don't Forget to click on the "FINISH" button

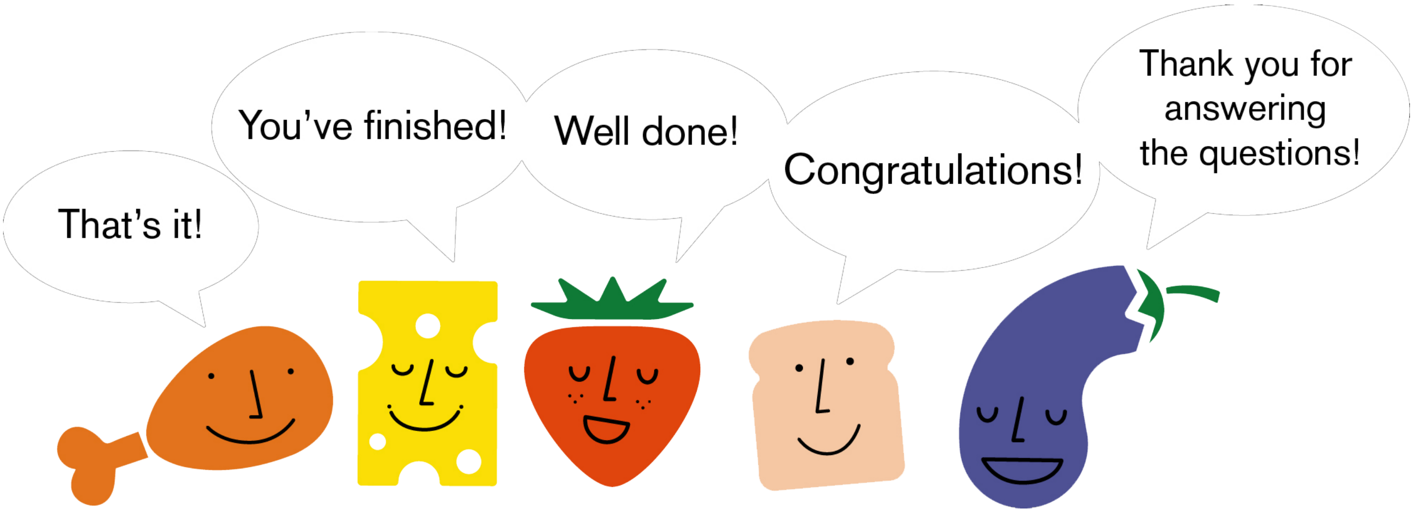

Supplement: Supplementary file 6 — Additional file 6. FEAST Student Evaluation [file 12889_2021_10302_MOESM6_ESM.pdf]
