# Supplementary material for: Evaluating OzHarvest’s primary-school Food Education and Sustainability Training (FEAST) program in 10–12-year-old children in Australia: protocol for a pragmatic cluster non-randomized controlled trial
Source: BMC Public Health. 2021 May 22;21:967. doi: 10.1186/s12889-021-10302-0 (PMC8140478; doi:10.1186/s12889-021-10302-0)
Supplement: Supplementary file 7 — Additional file 7. FEAST Teacher evaluation [file 12889_2021_10302_MOESM7_ESM.pdf]

# FEAST Teachers



WELCOME to the FEAST surveys!

Thank you for participating in the FEAST program.
Your feedback is invaluable as it helps us to improve the support and services OzHarvest provides to you, to deliver the FEAST program to your students.

On the following pages, there are some questions we would like to ask you.
If you do not understand a question, please contact the FEAST team at feast@ozharvest.org or (02) 8880 9791.

Your personal identity and information will be kept private and confidential.

If you would like to take part in the FEAST surveys, and you would like to help us by answering the questions, please click ‘NEXT'.

# Background

First Name

______________________________

Surname

______________________________

Email address

______________________________

Please enter your email address again:

______________________________

The emails entered did not match
Please ensure that the two email address entered above are exactly the same.

Would you like to receive a report of findings at the conclusion of the study?

◯ Yes
◯ No

What is your gender?

◯ Male
◯ Female
◯ Prefer not to say

What year do you teach?

◯ Year 5
◯ Year 6
◯ Year 5/6
◯ Other

Which grade do you teach? ________________________

How many students in your class? ________________________

How many students in your class participated in the FEAST program? ________________________

# FEAST teacher survey



Which type of FEAST training did you undertake?

◯ Face-to-Face
◯ Online
◯ Did not undertake training

The FEAST face-to-face training course prepared me to deliver the FEAST program in my classroom setting.

Please rate how strongly you agree/disagree with this statement.

◯ Strongly agree
◯ Agree
◯ Neutral
◯ Disagree
◯ Strongly disagree

The face-to-face Teacher Professional Development Training course delivered by OzHarvest was very effective.

Please rate how strongly you agree/disagree with this statement.

◯ Strongly agree
◯ Agree
◯ Neutral
◯ Disagree
◯ Strongly disagree

The FEAST online training course prepared me to deliver the FEAST program in my classroom setting.

Please rate how strongly you agree/disagree with this following statement.

◯ Strongly agree
◯ Agree
◯ Neutral
◯ Disagree
◯ Strongly disagree

The online Teacher Professional Development Training course delivered by OzHarvest was very effective.

Please rate how strongly you agree/disagree with this statement.

◯ Strongly agree
◯ Agree
◯ Neutral
◯ Disagree
◯ Strongly disagree

If you did not do any FEAST training, how easy/difficult was it to deliver the FEAST program in your classroom setting.

◯ Very easy
◯ Easy
◯ Neutral/Undecided
◯ Difficult
◯ Very Difficult

Did you teach the FEAST program on your own, or did you co-teach the FEAST program with another teacher/staff member from your school?

◯ Taught FEAST on my own
◯ Co-taught FEAST with another teacher/staff member from the school

How many FEAST lessons did you teach? __________________________________

How many cooking activities did you do with your class? __________________________________

Who co-taught the FEAST program with you?

◯ A teacher from my school
◯ A staff member from my school
◯ Other

How many FEAST theory lessons did they teach? __________________________________

Please tell us who the 'other' person was. __________________________________

How many FEAST cooking activities did they do? __________________________________

**Please rate the effectiveness of the FEAST resources provided, in aiding you to deliver the FEAST program.**

| | Did not receive resource | Very effective | Effective | Moderately effective | Slightly effective | Not effective |
|---|---|---|---|---|---|---|
| Online teacher portal | ◯ | ◯ | ◯ | ◯ | ◯ | ◯ |
| Online student portal | ◯ | ◯ | ◯ | ◯ | ◯ | ◯ |
| Student resources | ◯ | ◯ | ◯ | ◯ | ◯ | ◯ |
| FEAST unit of work | ◯ | ◯ | ◯ | ◯ | ◯ | ◯ |
| STEM lesson plans | ◯ | ◯ | ◯ | ◯ | ◯ | ◯ |
| FEAST practical guide | ◯ | ◯ | ◯ | ◯ | ◯ | ◯ |
| Kitchen Kit | ◯ | ◯ | ◯ | ◯ | ◯ | ◯ |
| Cold recipe book | ◯ | ◯ | ◯ | ◯ | ◯ | ◯ |
| Hot recipe book | ◯ | ◯ | ◯ | ◯ | ◯ | ◯ |
| Optional learning experiences | ◯ | ◯ | ◯ | ◯ | ◯ | ◯ |

| | |
|---|---|
| Did you use the FEAST 'Online Learning' resources in response to COVID-19? | ◯ Yes<br>◯ No |
| Please rate the effectiveness of the 'Online Learning' resources, in aiding you to deliver the FEAST program. | ◯ Did not receive resource<br>◯ Very effective<br>◯ Effective<br>◯ Moderately effective<br>◯ Slightly effective<br>◯ Not effective |

**Please rate how strongly you agree/disagree with the following statements...**

| | Strongly agree | Agree | Neutral | Disagree | Strongly disagree |
|---|---|---|---|---|---|
| The FEAST program content satisfactorily aligned with the identified Year 5 & 6 KLAs. | ○ | ○ | ○ | ○ | ○ |
| The FEAST program satisfactorily aligned with the cross-curriculum priority of sustainability. | ○ | ○ | ○ | ○ | ○ |
| I found the STEM lesson plans easy to implement in my classroom. | ○ | ○ | ○ | ○ | ○ |
| The FEAST program satisfactorily aligned with the general capabilities. | ○ | ○ | ○ | ○ | ○ |
| The FEAST program met my student's learning needs. | ○ | ○ | ○ | ○ | ○ |
| The FEAST website was easy for me to navigate. | ○ | ○ | ○ | ○ | ○ |
| The FEAST program was easy to integrate into my daily classroom routine. | ○ | ○ | ○ | ○ | ○ |

**Please rate how strongly you agree/disagree with the following statements...**

| | Strongly agree | Agree | Neutral | Disagree | Strongly disagree |
|---|---|---|---|---|---|
| My students found the FEAST activities easy to follow. | ◯ | ◯ | ◯ | ◯ | ◯ |
| My students found the FEAST resources easy to use. | ◯ | ◯ | ◯ | ◯ | ◯ |
| My students found the FEAST activities engaging. | ◯ | ◯ | ◯ | ◯ | ◯ |
| My students found the FEAST website easy to navigate. | ◯ | ◯ | ◯ | ◯ | ◯ |

**Please rate how strongly you agree/disagree with the following statements...**

| | Strongly agree | Agree | Neutral | Disagree | Strongly disagree |
|---|---|---|---|---|---|
| The FEAST program helped students to understand why it is important to be aware of food waste. | ○ | ○ | ○ | ○ | ○ |
| Students are able to understand which behaviours can reduce food waste in the home and at school. | ○ | ○ | ○ | ○ | ○ |
| Students have increased knowledge and understanding of food waste on a local level. | ○ | ○ | ○ | ○ | ○ |
| Students have increased knowledge and understanding of food waste on a global level. | ○ | ○ | ○ | ○ | ○ |

**Following completion of FEAST, please rate how strongly you agree/disagree with the following statements...**

| | Strongly agree | Agree | Neutral | Disagree | Strongly disagree |
|---|---|---|---|---|---|
| My students understand where food comes from. | ○ | ○ | ○ | ○ | ○ |
| My students understand how to make healthier food choices. | ○ | ○ | ○ | ○ | ○ |
| My students understand how to prepare and cook food. | ○ | ○ | ○ | ○ | ○ |
| My students understand how to minimise food waste. | ○ | ○ | ○ | ○ | ○ |
| My students are eating more fruits and vegetables. | ○ | ○ | ○ | ○ | ○ |
| My students are eating less 'junk' food. | ○ | ○ | ○ | ○ | ○ |

After completing the FEAST program, do you believe student food waste behaviours have decreased by:

◯ 0%, students waste the same amount of food
◯ 5%, students stopped a small amount of food going to waste
◯ 10%, students have reduced their food waste
◯ 20%, students are now food fighters and have reduced a lot of their food waste
◯ I don't know
◯ Other...

Did you create your class cookbook?

◯ Yes
◯ No

Can you please explain why you did not create a class cookbook?
Please tick as many categories as apply.

☐ Did not have time to create a class cookbook.
☐ Did not have skills to create a class cookbook.
☐ Students did not have time to complete their recipes.
☐ Students did not have the skills to complete their recipes.
☐ I/we decided to do a different activity.
☐ Not suitable for my class/group.
☐ Other

If you completed a different activity instead, please provide us with information about the activity you chose for your students.

__________________________________

Did your class do a class cookbook launch?

◯ Yes
◯ No

Who did you invite to the launch?
Please tick as many categories as apply.

☐ Only our class.
☐ Classes that participated in FEAST.
☐ Only Year 5 and 6 classes.
☐ Whole school.
☐ Parents/caregivers who helped with FEAST.
☐ Parents and Citizens (P&C) or Parents and Friends (P&F) from the whole school.
☐ Community volunteers that helped with FEAST.
☐ Other schools.
☐ Other.

What do you think were the benefits to your students and/or school?

__________________________________

On a scale of 0 to 10, how likely are you to recommend the FEAST program to others?

◯ 10, Extremely likely to Recommend
◯ 9
◯ 8
◯ 7
◯ 6
◯ 5
◯ 4
◯ 3
◯ 2
◯ 1
◯ 0, Not likely to Recommend

Were there any barriers in the classroom/school setting that impeded students undertaking the FEAST program? ◯ Yes ◯ No

Please describe the barriers in the classroom/school setting that, in your opinion, impeded students undertaking the FEAST program.

| | |
|---|---|
| Were there any external barriers from outside of the school environment that impeded students undertaking the FEAST program? | ◯ Yes ◯ No |

Please describe the external barriers from outside of the school environment that, in your opinion, impeded students undertaking the FEAST program.

Was cost a barrier to delivering the program? ◯ Yes ◯ No

Please explain how "cost" is a barrier to delivering the program at your school, and what steps are required to help overcome this issue?

How did you find the process of finding volunteers to help you with the practical components of FEAST?

Please tick as many answers, as apply.

- ☐ Simple
- ☐ Quick
- ☐ Frustrating
- ☐ Complicated
- ☐ Time consuming
- ☐ Other

Please explain what other difficulties you had in finding volunteers to help you with the practical components of FEAST.

What was your favourite aspect of the FEAST program?

What aspects of the program had the greatest impact on your students? And, why?

Where there any aspects of the program you would suggest to OzHarvest to modify for future FEAST programs?

Did you complete the FEAST Program risk assessment prior to delivering the program to your students?

◯ Yes
◯ No

Were any of your students harmed in the classroom setting over the course of the FEAST Program?

◯ Yes
◯ No

How many of your students were harmed in the classroom setting over the course of the FEAST Program?

______________________________
(Enter a Number)

What types of harms occurred to your students in the classroom setting over the course of the FEAST Program?

☐ Cut while using a kitchen knife
☐ Injured while using a grater
☐ Burn from an electric frying pan
☐ An allergic reaction
☐ Other

What "Other" type of harm occurred?

______________________________

Will you continue implementing the FEAST program in your classroom?

◯ Yes
◯ No

What motivates / inspires you to continue implementing the FEAST program in your classroom?

What are the reasons you would not continue to implement the FEAST program in your classroom?

Do you have any other feedback, queries or comments?
